# High-Mobility Group Box 1 Protein Signaling in Painful Diabetic Neuropathy

**DOI:** 10.3390/ijms21030881

**Published:** 2020-01-30

**Authors:** Vikram Thakur, Jayanarayanan Sadanandan, Munmun Chattopadhyay

**Affiliations:** Department of Molecular and Translational Medicine, Center of Emphasis in Diabetes and Metabolism, Texas Tech University Health Sciences Center El Paso, El Paso, TX 79912, USA; vikram.thakur@ttuhsc.edu (V.T.); jyns83@gmail.com (J.S.)

**Keywords:** Diabetes, neuropathy, high mobility group box1 (HMGB1), inflammation, neuropathy, glycyrrhizin

## Abstract

Diabetes is a global epidemic and more than 50% diabetic patients are also diagnosed with neuropathy, which greatly affects the quality of life of the patients. Available treatments are not always successful due to the limited efficacy and complications, such as addiction and dependency. Studies have implicated that high mobility group box1 (HMGB1) protein plays a crucial role in neuroinflammation and the development of neuropathic conditions. HMGB1 is a proinflammatory cytokine that can be released from necrotic cells in passive form or in response to inflammatory signals as an active form. HMGB1 is the ligand for the receptor for advanced glycation end products (RAGE), and toll-like receptors, (TLR)-2 and TLR4, which also indirectly activates C-X-C chemokine receptor type 4 (CXCR4). We investigated whether blocking of HMGB1 can reduce pain and inflammation in diabetic neuropathic animals to further understand the role of HMGB1 in diabetic neuropathy. Type 2 diabetic rats and mice were treated with natural inhibitor of HMGB1, glycyrrhizin (GLC) for five days/week for four weeks at a dose of 50 mg/kg per day by intraperitoneal injection. The animals were divided into three categories: naïve control, diabetic alone, diabetic with GLC treatment. All of the behavioral analyses were conducted before and after the treatment. The expression of inflammatory markers and changes in histone acetylation in the peripheral nervous system were measured by immunohistochemistry and Western blot analysis after the completion of the treatment. Our study revealed that TLR4, HMGB1, CXCR4, and Nod-like receptor protein 3 (NLRP3) levels were increased in the spinal and dorsal root ganglia (DRG) neurons of Type 2 diabetic mice and rats with painful neuropathy. GLC treatment inhibited the increases in TLR4, NLRP3, and CXCR4 expressions and improved the mechanical and thermal pain threshold in these animals. Immunohistochemical studies revealed that hyperglycemia mediated inflammation influenced HMGB1 acetylation and its release from the neurons. It also altered histone 3 acetylation in the microglial cells. The inhibition of HMGB1 by GLC prevented the release of HMGB1 as well as H3K9 acetylation. These findings indicate that the interruption of HMGB1 mediated inflammation could ameliorate diabetic neuropathy and might exhibit a unique target for the treatment.

## 1. Introduction

Pain is a frequent and debilitating consequence of diabetic neuropathy [1]. In diabetic patients, peripheral nerve dysfunction might arise from a complex pathophysiologic process, being related to the continuing hyperglycemic state. The overall extent of hyperglycemia correlates with the severity of neuropathy [2]. Even though the treatment costs that relate to the painful component are unclear [3], the burden of this complication is more than financial, as it affects the standard of health and comfort status of the patients [4,5,6,7]. Accordingly, there remains a clear unfulfilled medical demand for therapies that provide greater efficacy. Substantial evidence indicates that long-lasting inflammation and the initiation of the immune mediated nerve pathophysiology are closely involved in the development and maintenance of neuropathic pain. The present study evaluated the mechanisms for the HMGB1 release and neuroimmune activation in diabetic painful neuropathy.

High mobility group box 1 (HMGB1) is a ubiquitous DNA-binding non-histone nuclear protein that regulates gene expression. Under pathogenic conditions or stress, HMGB1 translocates to the cytoplasm and to the extracellular environment from the cells by discrete secretion pathways or passively through the apoptotic or necrotic cells. The released extracellular HMGB1 interacts with the receptor for advanced glycation end products (RAGE) or Toll-like receptor 4 (TLR4) to activate inflammatory pathways and stimulate inflammatory responses in the body. As it binds to its receptor, HMGB1 triggers the activation of nuclear factor-κB (NF-κB) to nucleus, which leads to secreting a number of proinflammatory cytokines, including interleukin-1 (IL-1), interleukin-6 (IL-6), and tumor necrosis factor-α (TNF-α) [8]. Additionally, HMGB1-mediated receptor binding initiates downstream signaling pathways with the phosphorylation of extracellular signal-regulated kinase (ERK), p38 mitogen-activated protein kinase (p38MAPK), c-Jun N-terminal kinase (JNK), and myeloid differentiation factor-88 (MyD88) [9]. This positive feedback loop contributes to the release of further proinflammatory cytokines. Epigenetic enzymes, such as histone acetyltransferases (HATs), can acetylate nuclear HMGB1 by cyclic-AMP regulatory element binding protein (CREB)-binding protein (CBP) and p300/CBP-associated factor (PCAF) [10,11], and histone deacetylases (HDACs) particularly HDAC1 and HDAC4, can deacetylate HMGB1 [12]. The acetylated HMGB1 might translocate from nucleus to the cytoplasm, and might further release to the extracellular space to activate the receptors of the additional immune cells [13]. Continuing evidence clearly suggests that HMGB1 can act as pronociceptive agent in the maintenance of somatic and visceral pain [14,15]. Earlier studies have also validated that peripheral HMGB1 can act as pronociceptive agent in rodents when inflammatory hyperalgesia was induced by intraplantar administration of lipopolysaccharide [16], as well as in bladder pain with cyclophosphamide-induced cystitis [15]. The pronociceptive role of HMGB1 in the dorsal root ganglia (DRG) and spinal cord has been further confirmed in the inflammatory pain mouse model of collagen antibody-induced arthritis [14] and in rodents with surgical nerve injury induced neuropathy [17,18,19]. Previous studies have also revealed that chemokine C-X-C motif ligand 12 (CXCL12) and its receptor chemokine C-X-C motif receptor 4 (CXCR4) play crucial roles in neuronal and glial mechanisms in the development and maintenance of pain [20,21,22]. Our study also revealed an increase in CXCR4 expression in the DRG of diabetic animals with painful neuropathy. The release of HMGB1 from nucleus of DRG and spinal cord neurons and its translocation to the cytoplasm may involve post-translational modifications, such as acetylation, methylation, or phosphorylation. Further release of HMGB1 into the extracellular environment of the cell might also comprise of inflammasome activation. HMGB1 can also activate CXCR4 via forming a heterocomplex with CXCL12. However, it is not evident whether HMGB1 participates in diabetic neuropathic pain through these mechanisms that may identify a novel therapeutic approach. The outcomes of this study were confirmed in in vivo (Type 2 diabetic rat and mice) and in vitro (F11 DRG cell line) models.

Glycyrrhizin, which is an extract of licorice root, is a triterpenoid saponin glycoside used as a natural sweetener and it has been described with potential immunomodulating, anti-inflammatory, hepato- and neuro-protective, and antineoplastic activities [23,24,25,26]. Previous studies have shown that glycyrrhizin suppresses HMGB1 expressions and mitogenic activities, and impedes its DNA-binding function. Nuclear magnetic resonance and fluorescence studies have demonstrated that glycyrrhizin directly binds to HMGB1 [27]. In the current study, we examined whether glycyrrhizin mediated inhibition of HMGB1 is beneficial in alleviating pain in diabetic neuropathy. To be secreted, HMGB1 is post-translationally acetylated. Monocytes and macrophages mediate the active secretion of HMGB1, which plays an important role in acetylation. The acetylation of lysine residues in HMGB1 is essential for its route to the cytoplasm (8). This present study shows increased histone 3 lysine 9 (H3K9) acetylation in the non-neuronal cells, which was ameliorated by glycyrrhizin treatment. The increases in HMGB1 in the DRG and spinal cord dorsal horn neurons and release of HMGB1 may contribute to the maintenance of painful neuropathy in the diabetic animals. Our study demonstrates that treatment with glycyrrhizin alleviated the pain behaviors by blocking HMGB1 release from the neuronal cells and impeded histone 3 lysine 9 (H3K9) acetylation in the non-neuronal immune cells with a reduced expression of inflammatory mediators as well as possible association of c-Jun N-terminal kinase (JNK) and CXCR4 mediated pathway.

## 2. Results

### 2.1. Type 2 Diabetic ZDF Animals Exhibited a Significant Change in Mechanical and Thermal Hyperalgesia after Treatment with HMGB1 Inhibitor

**Figure 1:**
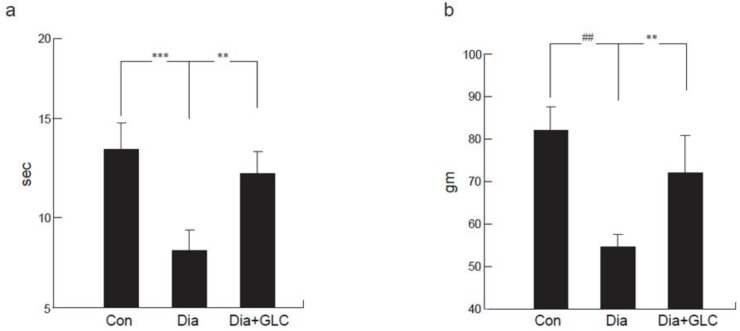
Alterations in mechanical and thermal pain behaviors in type 2 diabetic animals following treatment with Glycyrrhizin (GLC). (**a**) Thermal withdrawal latency (Hargreaves test) exhibited a reduction in latency in response to unpleasant thermal stimulus in diabetic animals when compared with control animals (*p* < 0.001). GLC treated animals showed significant amelioration in thermal hyperalgesia compared to diabetic only animals (*p* < 0.01). (**b**) Diabetic animals exhibited significant mechanical hyperalgesia (Randall-Selitto) compared to control animals (*p* < 0.0001). Animals treated with GLC showed significant alleviation of mechanical hyperalgesia compared to diabetic only animals (*p* < 0.01). Con: naïve control; Dia: diabetic only group; Dia+GLC: diabetic group treated with glycyrrhizin. The data presented in the graph indicates mean ± SEM, *n* = 6–8 per group. ** *p* < 0.01; *** *p* < 0.001; ## *p* < 0.0001.

Zucker diabetic rats exhibited thermal hyperalgesia, which was determined by a decrease in withdrawal latency, measured in seconds (sec), when exposed to noxious thermal stimuli on a modified Hargraves apparatus (control 14.1 ± 1.9 sec; diabetic 7.62 ± 1.3 sec; *p* < 0.001) as compared to the control animals (Figure 1a). ZDF animals treated with GLC showed significant alleviation in thermal hyperalgesia (13.6 ± 1.5 sec; *p* < 0.01). The Randall–Selitto method of mechanical hyperalgesia was used to measure paw pressure in grams (gm). ZDF animals showed a significant decrease in hind-paw withdrawal threshold when compared to control animals (82.1 ± 8.4 gm vs 54.2 ± 5.4 gm; *p* < 0.0001) measured three days post-treatment. To determine whether increased HMGB1 level in peripheral nervous system is responsible for the painful neuropathy at four weeks after diabetes in animals, GLC was administered for five days a week for four weeks at a dose of 50 mg/kg per day I.P. ZDF animals that were treated with GLC showed significant alleviation of mechanical hyperalgesia (71.2 ± 9.1 gm; *p* < 0.01; Figure 1b).

### 2.2. Increased Neuroinflammation in DRG of ZDF Rats with Painful Neuropathy was Ameliorated by Glycyrrhizin Treatment

**Figure 2:**
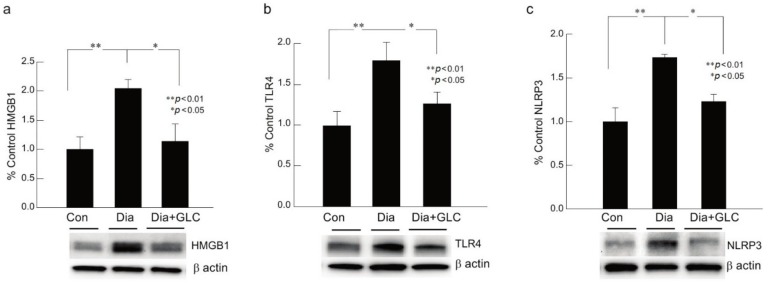
Glycyrrhizin treatment ameliorated neuroinflammation in DRG of animals with PDN. (**a**–**c**) Type 2 diabetic animals with painful neuropathy demonstrated a significant increase in HMGB1, TLR4 and NLRP3 in DRG at eight weeks after hyperglycemia as compared to their control counterparts. Treatment with GLC confirmed alleviation of neuroinflammation with decreased expressions of NLRP3, TLR4, and HMGB1 compared to the diabetic animals with no treatment. Western blots are representation of one sample from each group. There are no significant differences between control vs diabetic+GLC treated groups. Con: naïve control; Dia: diabetic only group; Dia+GLC: diabetic group treated with glycyrrhizin. The data presented in the graph indicates mean ± SEM, *n* = 6–8 per group. * *p* < 0.05; ** *p* < 0.01.

ZDF animals with Type 2 diabetic painful neuropathy revealed a significant increase in NLRP3, HMGB1, and TLR4 in DRG at eight weeks after hyperglycemia when compared to their control counterparts. Diabetic animals, four weeks after the onset of hyperglycemia, treated with HMGB1 inhibitor GLC for four weeks, demonstrated an alleviation of neuroinflammation with decreased expressions of NLRP3, TLR4, and HMGB1 when compared to the diabetic animals with no treatment, as shown by Western blot analysis, which is concomitant with decreased pain behavior in GLC treated diabetic animals as compared to diabetic animals with no treatment (Figure 2a–c).

### 2.3. Glycyrrhizin Prevents Hyperglycemia-Induced HMGB1 Cytoplasmic Relocation in DRG and Extracellular Release in Spinal Cord Neurons as well as Alleviates TRPC6 Expression

**Figure 3:**
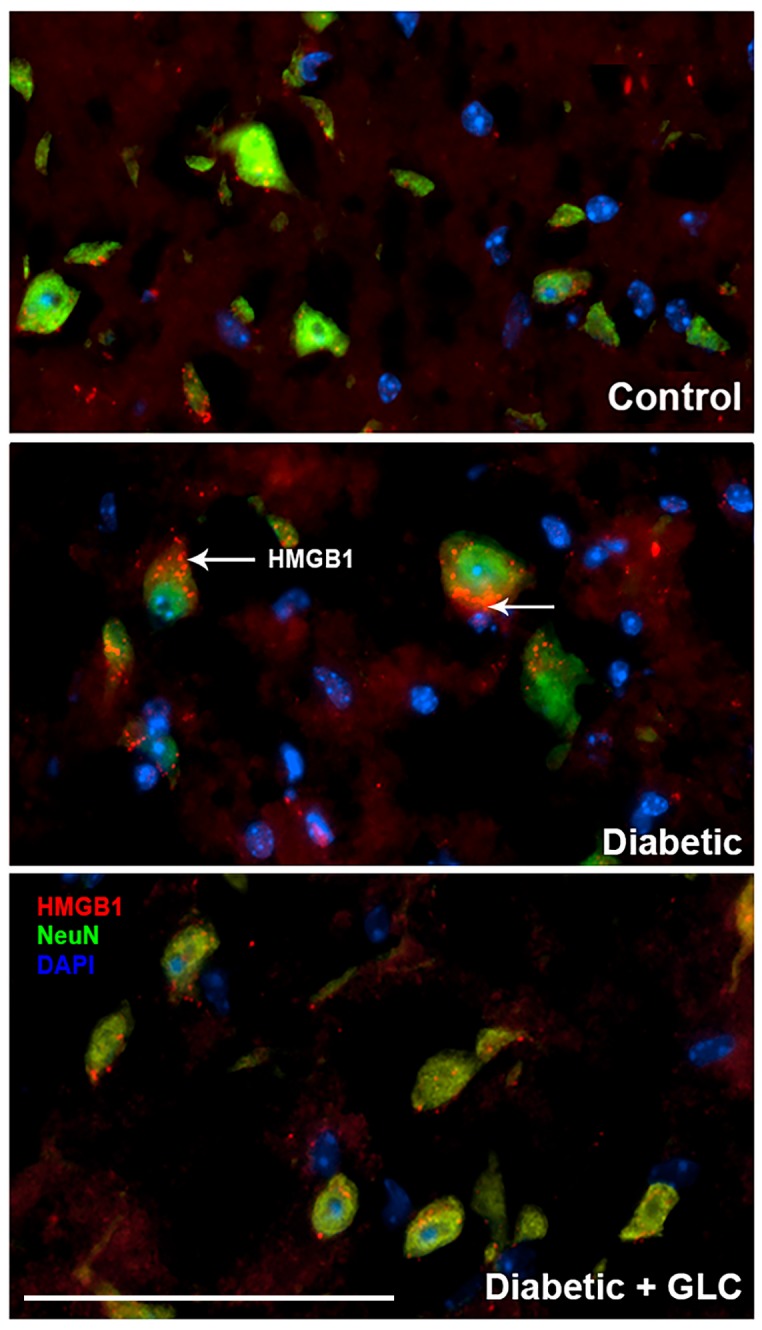
GLC treatment inhibits hyperglycemia-induced HMGB1 cytoplasmic and extracellular release in spinal cord neurons of Type 2 diabetic mice. Immunohistochemical studies of HMGB1 expression in the spinal cord dorsal horn of ob/ob mice show cytoplasmic and extracellular HMGB1 release from neuronal cells and GLC treatment blocked the release. NeuN antibody, biomarker for neuron shows in green and DAPI (4’,6-diamidino-2-phenylindole), a blue-fluorescent dye stains DNA in the nucleus. Bar = 200 µM.

**Figure 4:**
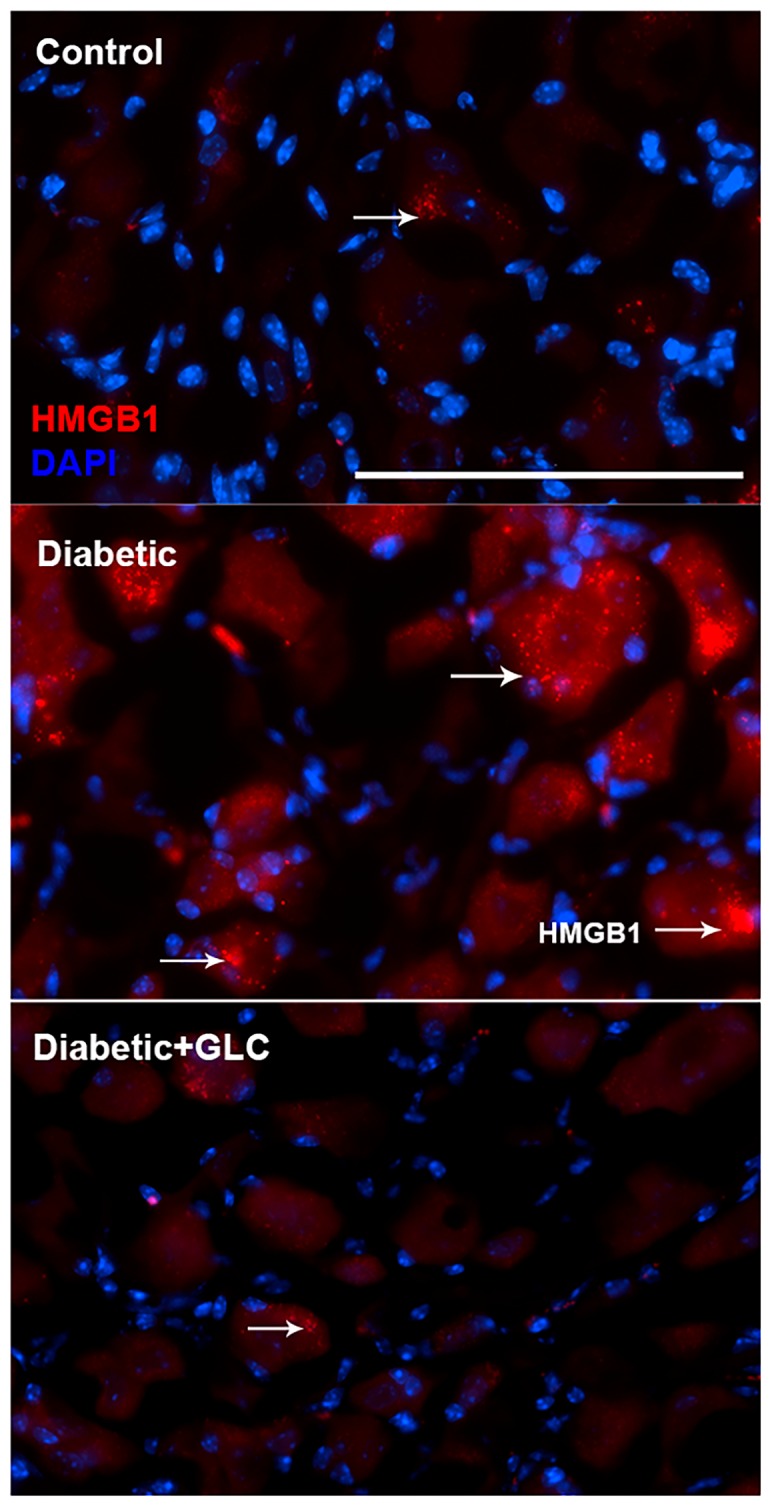
Hyperglycemia-induced cytoplasmic release of HMGB1 in DRG neurons of type 2 diabetic rats was hindered by GLC treatment. Immunohistochemical analysis indicated enhanced HMGB1 expression in sensory neurons and its translocation into the cytoplasm. GLC treatment abolished the increase in HMGB1 cytoplasmic release in DRG neurons of Zucker diabetic fatty (ZDF) rats. Bar = 200 µM.

**Figure 5:**
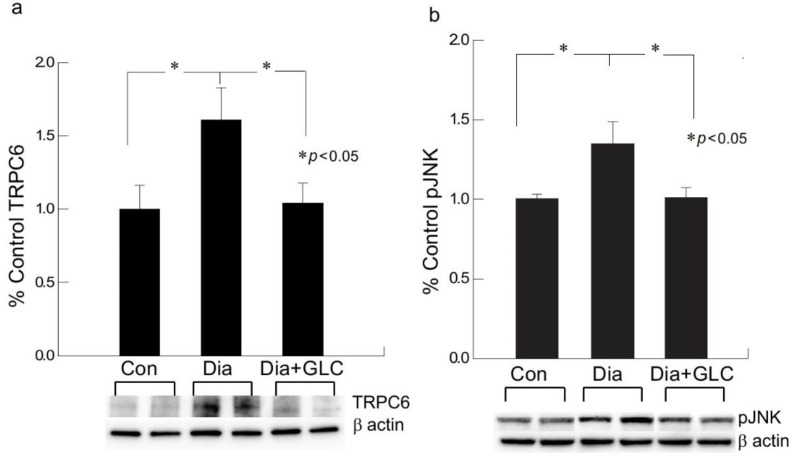
Increased expression of TRPC6 and activation of c-Jun N-terminal kinase (JNK) in spinal cord neurons of type 2 diabetic animals was impeded by GLC treatment. (**a**,**b**) Type 2 ob/ob diabetic mice showed increased expression TRPC6 in the spinal cord neurons whereas activation of JNK was observed in the spinal cord of ZDF rats. GLC treatment alleviated the expression of TRPC6 and altered the JNK activation in both animal models. There are no significant differences between control vs diabetic+GLC treated groups. Con: naïve control; Dia: diabetic only group; Dia+GLC: diabetic group treated with glycyrrhizin. * *p* < 0.05.

We performed behavioral studies to confirm that Type 2 diabetic mice and rats show similar experimental outcomes which demonstrated similar results as ZDF rats. The immunohistochemical studies of HMGB1 in the spinal cord dorsal horn of ob/ob mice exhibited hyperglycemia mediated HMGB1 release, predominantly from neuronal cells, and treatment with GLC inhibited the release of HMGB1 from the neuronal cells to the cytoplasm or to the extracellular space (Figure 3), which was also validated by Western blot analysis and the immunohistochemistry of the DRG of ZDF rats with painful diabetic neuropathy (PDN) (Figure 2 and Figure 4). Here, we report that Transient Receptor Potential Canonical (TRPC) channel 6 expression in the spinal cord was up-regulated in ob/ob diabetic mice eight weeks post-diabetes. Furthermore, the inhibition of HMGB1 in the spinal cord blocked the increase of TRPC6 and hyperalgesia without any change in the blood glucose levels (Figure 5a).

### 2.4. GLC Inhibited Hyperglycemia Induced Activation of JNK in Spinal Cord and CXCR4 Expression in DRG

**Figure 6:**
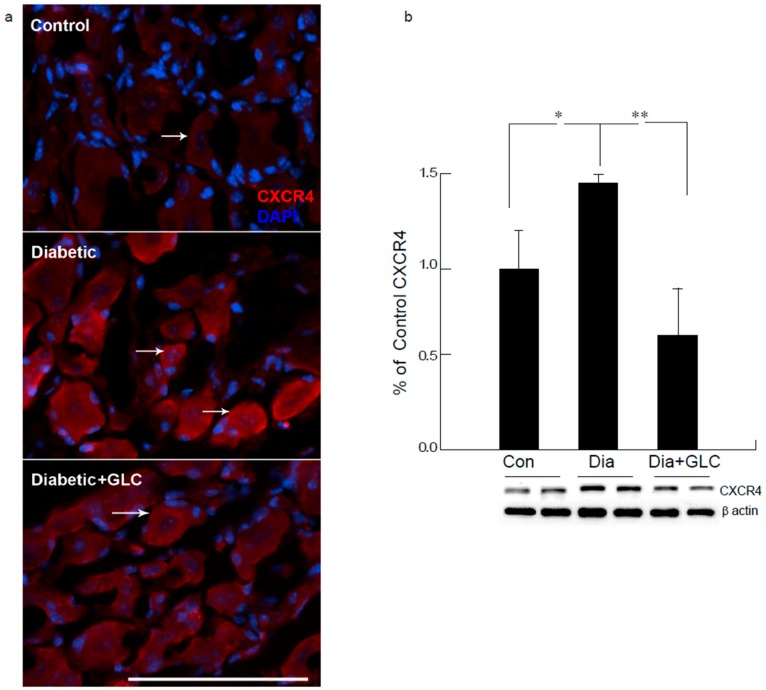
Increased expression of CXCR4 in DRG neurons of ZDF diabetic animals was blocked by glycyrrhizin. Type 2 diabetic DRG neurons demonstrated increased expression of CXCR4 as displayed by immunohistochemical (**a**) and Western blot analysis (**b**). Treatment with GLC reduced the CXCR4 expression in DRG neurons. Bar = 200 µM. Con: naïve control; Dia: diabetic only group; Dia + GLC: diabetic group treated with glycyrrhizin. * *p* < 0.05; ** *p* < 0.01.

Western blot analysis of the spinal cord dorsal horn of ZDF rats demonstrated that c-Jun N-terminal kinases (JNK) were significantly activated when compared to the control animals and treatment with GLC revealed the alteration of the activation of JNK (Figure 5b), which further confirmed the possible association of JNK pathway. CXCR4 is widely present in the sensory neurons of the peripheral nervous system (PNS) as well as in the central nervous system (CNS) while also contributing to the pain signaling of both systems. Our study revealed that CXCR4 is increased in DRG neurons of ZDF diabetic rats, as validated by immunohistochemistry and Western blot analysis (Figure 6). Treatment with GLC reduced CXCR4 expression in DRG neurons.

### 2.5. Global Acetylation of H3K9 was Increased in the Spinal Cord Dorsal Horn of Diabetic Animals as well as in the Hyperglycemic DRG Neuronal Cell Line

**Figure 7:**
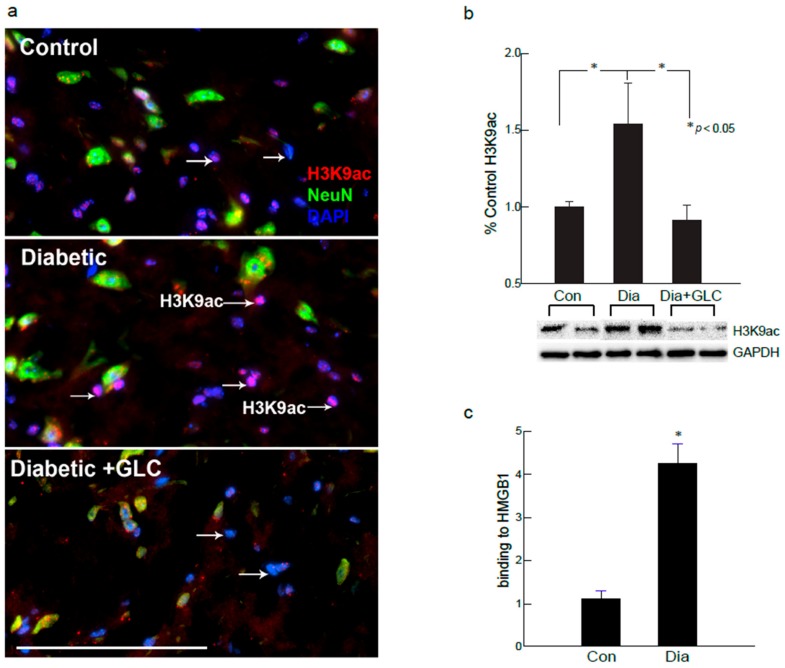
H3K9ac-dependent spinal HMGB1 expression in diabetic animals with painful neuropathy. (**a**) Diabetic ob/ob mice increased nuclear H3K9ac activity in non-neuronal glial cells of spinal cord. Treatment with GLC ameliorated the H3K9 acetylation in diabetic spinal cord neuronal and glial cells. (**b**) This increase was confirmed by western blot analysis of spinal cord of ZDF rats which also demonstrated an alleviation of H3K9ac expression in the GLC treated animals. (**c**) Further confirmation of results with ChIP analyses revealed that the hyperglycemia mediated increased acetylation of H3K9 is associated to HMGB1 promoter in the spinal dorsal horn of ZDF animals. Bar = 200 µM. Con: naïve control; Dia: diabetic only group; Dia+GLC: diabetic group treated with glycyrrhizin. * *p* < 0.05.

Changes in the global expression of H3K9 acetylation (H3K9ac) protein were examined in the spinal cord of ob/ob mice. H3K9ac immunoreactivity appears in the neurons and as well as in the non-neuronal profiles to explore the epigenetic involvement in the spinal cord neurons and glial cells of diabetic animals with painful neuropathy (Figure 7). Positive immunofluorescence of H3K9ac was predominately expressed in the non-neuronal cells as well as moderately in the medium- and small-sized neurons. The proportion of H3K9ac-positive neuronal cells did not significantly differ from that of the control group versus the GLC treatment group. In contrast, the percentage of H3K9ac-positive neurons increased significantly in the diabetic group with painful neuropathy (*n* = 3 per group, *p* ≤ 0.05) 6–8 weeks after diabetes as compared to the GLC treatment group. These results provide robust evidence of epigenetic modulation in the spinal cord dorsal horn of the diabetic animals.

### 2.6. H3K9ac-Dependent Spinal HMGB1 Expression in Diabetic Animals with Painful Neuropathy

Hyperglycemia has been implicated in increased HMGB1 expression in sensory neurons and endothelial cells [28,29,30,31]. Therefore, we explored whether the acetylation of spinal HMGB1 was altered in diabetic neuropathy. Six to eight weeks after hyperglycemia, ob/ob diabetic animals exhibited increased acetylation of HMGB1 in the neuronal and non-neuronal cells as compared the control group (Supplementary Figure S1), and these mice also showed increased nuclear H3K9ac activity in non-neuronal glial cells (Figure 3). We next examined the relationship between the subsistence of HMGB1 and H3K9ac to confirm that increased H3K9 acetylation might promote acetylated HMGB1 release from neurons. The results of ChIP analyses further confirmed the hyperglycemia mediated increased acetylation of H3K9 and its association to the HMGB1 promoter that up-regulated HMGB1 expression in spinal dorsal horn of ZDF animals six to eight weeks after hyperglycemia (Figure 7c).

### 2.7. Hyperglycemic DRG Neuronal Cell Line Exhibited Increased HMGB1 Expression with Increased Global Acetylation of H3K9 

An immortalized F11 DRG neuronal cell line was used to confirm whether hyperglycemia could influence the expression of HMGB1 and alter the global acetylation of H3K9 in DRG neuronal cells. After overnight hyperglycemic exposure (16–18 h), the cells were harvested to evaluate the expression of HMGB1, receptor for advanced glycation end products (RAGE), and compared between normoglycemic and hyperglycemic conditions. Hyperglycemic neuronal cells exhibited increased HMGB1 and RAGE expression (Supplementary Figure S2). H3K9 acetylation under the hyperglycemic condition was also confirmed, which demonstrated that overnight exposure to 25 mM glucose increased H3K9 acetylation, as demonstrated by immunocytochemical analysis (Supplementary Figure S3).

## 3. Discussion

Neuropathy is a significant complication of diabetes and it is difficult to treat, as there are not many analgesic agents that work well on neuropathic pain without serious side effects. Many studies have found that inflammation might play a role in painful diabetic neuropathy. The advanced glycation end products (AGE) pathway mediated release of inflammatory cytokine HMGB1 is of interest in our study. HMGB1 is a non histone DNA binding nuclear protein hat plays a role in DNA repair, replication, and gene transcription [32]. The importance of HMGB1 in nucleus is not fully understood, whereas recent studies have shown that extracellular and cytoplasmic HMGB1 controls prototypical danger signal during inflammatory and repair responses. The passive release of HMGB1 from damaged cells or activated immune cells following infection, injury, and sterile inflammation also demonstrates the regulation of inflammatory responses [33,34,35,36], which can also be mediated through inflammasomes, large caspase-1-activating protein complexes, including NLRP3 [37], and, more importantly, these cytoplasmic release of HMGB1 from activated glia or injured neurons can also act as a cytokine [38,39]. Earlier studies have shown that HMGB1 stimulates the release of IL-1α, IL-1β, and IL-6 in cultured human primary macrophages [40]. HMGB1 might initiate downstream signaling through the receptor for advanced glycation end products (RAGE), and via toll-like receptors, TLR2 and TLR4. TLR4 is vastly expressed by microglial cells, and it has also been identified in astrocytes, neurons, and neural progenitor cells [41,42,43]. In our study, we found that the TLR4, HMGB1, and NLRP3 levels were up regulated in Type 2 diabetic rat models with painful neuropathy and blocking of HMGB1 by natural inhibitor glycyrrhizin treatment decreased the expression of TLR4, HMGB1, and NLRP3, as well as ameliorated mechanical and thermal pain threshold.

Extracellular release of HMGB1 in disease pathogenesis, such as sepsis, arthritis, colitis, and ischemia reperfusion, has validated that the inhibition of HMGB1 significantly reduces the severity of many of these conditions [35,44,45,46]. To be secreted, HMGB1 must undergo post-translational modification, like histone acetylation. Acetylation plays an important role for the active release of HMGB1 from nucleus to cytoplasm by neurons and microglial cells. The translocation of HMGB1 to the cytoplasm might deduce acetylation of lysine residues in histone 3 in the spinal glial cells [47]. Our study demonstrates that release of HMGB1 by neurons in the spinal cord provides a plausible basis of an endogenous inflammatory mediator that can modulate adjacent neuronal and glial signals (Figure 3 and Figure 6). Recent evidence also suggests that cortical neuron mediated HMGB1 signaling might contribute to rapid changes in neuronal excitability that may lead to the development of neuropathic pain states [31,48]. Our study establishes that diabetic animals with painful neuropathy exhibited increases in HMGB1 shuttling from the nucleus to the cytoplasm, but this phenomenon was almost completely altered after the treatment with GLC, thereby alleviating the mechanical and thermal hyperalgesia in these animals. The significant increase in c-Jun N-terminal kinases (JNK) in diabetic animals when compared to control animals and the amelioration of this activation post-treatment with GLC points to the role of HMGB1 in PDN and its possible association with the JNK pathway (Figure 5). Previous studies with normal human bronchial epithelial cells showed that HMGB1-induced activation of JNK and NF-κB were attenuated by the pretreatment with JNK inhibitor SP600125, which indicates that HMGB1 induced inflammation is mediated through the RAGE/JNK/NF-κB pathway [49]. Another study also supported this observation and substantiated that HMGB1 was secreted from TNF-α-induced 3T3-L1 adipocytes through JNK signaling [50].

These observations denote the significance of a mechanistic elucidation of HMGB1 release from neurons and activated immune cells and understanding of the downstream signaling pathways that control these processes. Histone modifications play a substantial role in gene regulation. H3K9ac is usually linked with transcription activation, which consequently impacts the gene expression. Studies have shown that the scarcely expressed gene promoters have minimal H3K9ac signal, while an enhanced acetylation signal is found in the significantly high expressed promoters [51], which indicates a further explicit link between H3K9ac and gene expression level. Nonetheless, acetylation is very much required for the active secretion of HMGB1 by macrophages as well as microglial cells. For the translocation of HMGB1 to the cytoplasm from the nucleus, the acetylation of lysine residues is essential [47]. Stimulation by inflammatory mediators, such as IL-1β and TNFα, is required for the transfer of HMGB1, which was confirmed by the western blot analysis of the spinal cord dorsal horn in control, diabetic and diabetic GLC animals (Supplementary Figure S1). We next examined the relationship between the availability of HMGB1 and H3K9ac, as our immunohistochemical studies showed increased H3K9ac activity in spinal dorsal horn along with increased HMGB1 release from neurons. Chromatin immunoprecipitation (ChIP) analyses indicated that hyperglycemia significantly increased the levels of H3K9ac at the promoter region of HMGB1 in the dorsal horn of diabetic animals as compared with the control animals (Figure 7). All through the human genome, H3K9ac is positioned near the transcription start sites [52,53]. Studies have shown that H3K9ac levels around individual promoters frequently do not show the normal genome-wide distribution. The H3K9ac signal is weak in the poorly expressed gene promoters, while significantly higher expressed promoters have an enhanced acetylation signal [51].

The transient receptor potential canonical channel TRPC6 showed increased expression in diabetic spinal cord neurons, whereas it was decreased in the GLC treated animals. The role of TRPC6 in brain development is under defined, whereas it has been established that TRPC6 controls cytosolic, endoplasmic reticulum, and mitochondrial Ca^2+^ levels in neural cells. Most isoforms of TRPC channels are expressed in neuronal cells, whereas their physiological and ion channel properties and mode of activation are quite discrete. Moreover, TRPCs also regulate neuroinflammation, synaptic plasticity, and potentiation [54]. Studies have shown that, in dorsal root ganglion neurons, TRPC6 influences nociceptive mechanical hyperalgesia induced by inflammatory mediators in conjunction with TRPV4 and mediates primary afferent nociceptor sensitization [55]. Hence, understanding the detailed involvement of TRPC6 and HMGB1 in painful neuropathy would presumably reveal possibilities for therapeutic interventions for this devastating condition.

The presence of CXCR4 in the sensory neurons of both PNS and CNS is important in pain signaling. The pronociceptive role of CXCL12/CXCR4 axis is shown to be involved for the development and maintenance of neuropathic pain. The intrathecal injection of CXCL12 demonstrated an increased phosphorylation of the ERK, JNK, and p38 MAPK pathways in the spinal dorsal horn of naïve rats, and the pretreatment of CXCR4 antagonist, AMD3100 reversed this activation [56]. Recent studies on the pathophysiological mechanisms of CXCL12/CXCR4 axis in the pathogenesis of pain by various nerve injury and neuropathy models, including diabetic neuropathy, demonstrate the axis as a promising pharmacological target. Our study shows that the release of HMGB1 might be involved in the activation of JNK pathways and blocking the release of HMGB1 by GLC may impede pain through alterations in CXCR4 mediated pain pathways, which could be an advantageous therapeutic approach for diabetic neuropathy. This unique study also confirmed that, irrespective of Type 2 diabetic rat or mice model as well as hyperglycemic cell culture model, hyperglycemia increased the expression of HMGB1 and H3K9ac with alterations in inflammatory mediators.

## 4. Materials and Methods

### 4.1. Experimental Design

The Zucker diabetic fatty (ZDF; Charles River, Wilmington, MA, USA) rat is a spontaneous Type 2 diabetic animal model, in which hyperglycemia primarily establishes at about 8–9 weeks of age. Male animals were used in the study and housed in pairs in a SPF facility with the recommended light/dark cycle, humidity, and temperature. For the duration of the whole experiment, ZDF rats were given Purina #5008 lab diet and water, ad libitum. Ten weeks old rats with greater than 300 mg/dL blood glucose level were considered to be diabetic in the study (Supplementary Figure S4). The blood glucose levels were evaluated while using OneTouch Ultra glucose meter (LifeScan, Inc., Milpitas, CA, USA) once a week in the morning. Obese mice, homozygous for spontaneous leptin mutation, *Lep^ob^* (ob/ob; Jackson labs, Bar Harbor, ME, USA), were also included in the study to confirm the outcome. Experiments were carried out in accordance with approved institutional animal care and use protocols (IACUC, TTUHSC).

Four weeks after the onset of diabetes, 14 weeks old animals were injected HMGB1 inhibitor Glycyrrhizin (GLC) for five days a week for four weeks at a dose of 50 mg/kg per day I.P. The animals were categorized into three groups: naïve control, diabetic alone, diabetic with GLC treatment. Each group consists of 8–10 animals. All of the behavioral analyses were carried out in the early morning before and after the treatment by an observer blinded to the treatment group. The expression of inflammatory markers and changes in histone acetylation in the peripheral nervous system of the diabetic animals were measured by immunohistochemistry and Western blot analysis after the completion of the treatment. At the completion of treatment regimen, eighteen weeks old animals were euthanized according to the recommendation of the AVMA Panel on Euthanasia and IACUC policy to harvest tissue samples, as proposed in the approved IACUC policy.

Ethics approval and consent to participate: Experiments were performed in compliance with approved institutional animal care and use protocols (IACUC, TTUHSC El Paso, TX) under the protocol #14035 (30.12.2019) and 14014 (24.09.2019).

### 4.2. Behavioral Studies

#### 4.2.1. Thermal Hyperalgesia

Thermal hyperalgesia was measured by evaluating the latency of the paw withdrawal from an unpleasant thermal stimulus while using a modified Hargreaves apparatus [57]. The animals were acclimated for 3–5 days before the test on a glass plate at 30 °C in separate enclosures for 20–30 mins each day. On the day of the testing, animals were accustomed for 30 min period in the enclosures. A beam of radiant light at 50 °C was presented to the plantar surface of the hind paw through the glass floor. A timer with a cut-off time of 20 sec activated the light, and it turned off with motion of the paw withdrawal. The tests were repeated three times by a blinded observer and at least 10 min intervals were given between the repeats.

#### 4.2.2. Mechanical Hyperalgesia

Mechanical hyperalgesia was measured while using an analgesimeter (Ugo Basile, Comerio, VA, Italy) to assess nociceptive threshold as described by Randall and Sellito [58]. A 1 mm cone-shaped plastic tip was placed between the third and fourth metatarsus and an amplifying pressure was employed on to the dorsal surface of the hind paw. The weight was applied until the rat retracted the paw from the pressure or a 200 gms cutoff pressure was reached. Three consecutive steady values, expressed in grams, were taken to determine the mechanical pain threshold, and the means were calculated by a blinded observer.

### 4.3. Western Blot

Lumbar DRG 4-6 and spinal cord dorsal horn from each rat and mouse were harvested fresh and homogenized with lysis buffer and western blot technique was employed, as described in our previous work [59]. The primary antibodies anti-HMGB1, RAGE (1:500; Cell Signaling, Danvers, MA, USA), HMGB1ac (1:500; Lifespan Biosciences, Seattle, WA, USA), TLR4, pJNK (1:500; Santa Cruz Biotechnology, Santa Cruz, CA, USA), NLRP3 (1:400; Novus Biologicals, Littleton, CO, USA) TRPC6 (Millipore-Sigma, St. Louis, MO, USA), CXCR4 (Thermo Fisher Scientific, Waltham, MA, USA), and secondary antibody horseradish peroxidase-conjugated anti-rabbit IgG or anti-mouse IgG (1:5000; Amersham, Piscataway, NJ, USA) were used according to the protocol and imaged with ECL (Pierce Biotechnology Inc., Rockford, IL, USA). β-actin (1:2000; MilliporeSigma, St. Louis, MO, USA) was used as a loading control. A PC-based image analysis system (ChemiDoc XRS System, Bio-Rad Laboratories, Hercules, CA, USA) determined the intensity of each band by quantitative chemiluminescence and the data were normalized to the respective level of β-actin. Data are expressed as mean SEM (*n* = 3–5 animals per group).

### 4.4. Chromatin Immunoprecipitation (ChIP)

ChIP was performed while using EpiQuik Tissue ChIP Kit (Epigentek, Farmingdale, NY, USA) consistent with a modified manufacturer protocol. The dissected ZDF spinal cord samples were excised into smaller pieces (1–2 mm^3^). The sliced and crushed samples were incubated with fresh 1% paraformaldehyde to cross-link proteins to DNA on a shaker for 15–20 min at 25–30 °C. Subsequently, instructions from the protocol were followed accordingly. Chromatin fragments were generated from the sheared lysates procedure by sonication. The input control for PCR was saved from the sonicated chromatin. The chromatin was then immunoprecipitated for 2 h at RT with antibody against H3K9ac (Epigentek, Farmingdale, NY, USA) or control IgG. The protein—DNA immunocomplexes were precipitated while using spin columns and several steps were followed, as instructed. The purified DNA was isolated with specific immunoprecipitate or with negative control IgG and used as a template for PCR to amplify the HMGB1 promoter sequences. A 197-base pair fragment were amplified while using the ChIP primer sequences: 5′-CTCCAGGAAACGGCTTTGTA-3′ and 3′-TCCACAGAGTTAGTTCCAGAGGA-5′ corresponding to the core HMGB1 promoter.

### 4.5. Immunohistochemistry

Rats and mice were perfused with 0.9% NaCl and Zamboni’s fixative transcardially [60]. Lumber DRG 4 to 6 and spinal cord dorsal horn were harvested and post-fixed with Zamboni’s fixative for 2 h. Cryoprotection was achieved while using 30% sucrose in phosphate buffered saline (PBS) overnight. The tissues were then sectioned and washed with PBS, incubated with blocking solution (PBS with 1% normal goat serum and 0.3% Triton X-100) for 1 h, and further washed before being incubated with the primary antibody anti-HMGB1 (1:400; Cell Signaling, Danvers, MA, USA), H3K9ac (1:500; Epigentek, Farmingdale, NY, USA), CXCR4 (1:2000; ThermoFisher Scientific, Waltham, MA, USA), NeuN (1:800; Millipore-Sigma, St. Louis, MO, USA) and GFAP (1:1000; Santa Cruz Biotechnogy, Santa Cruz, CA, USA) overnight at 4 °C. Following three washes, Alexa Fluor 594 goat anti-rabbit IgG and 488 goat anti-mouse IgG at a concentration of 1:1000 were used as secondary antibodies (Molecular Probes, Eugene, OR, USA) for one hour incubation, washed three times, and stained with DAPI. The slides were finally mounted with Fluoromount G (Electron Microscopy Sciences, Fort Washington, PA, USA).

### 4.6. Comparative Analysis

The immunostained digital images of each sample were captured with a Nikon Eclipse Ni-E microscope and analyzed while using the NiS Elements computer-based image analysis system (Nikon Instruments Inc., Melville, NY, USA). The image analysis software was used to measure the intensity of the immunostained neurons and glial cells of the spinal cord and DRG. An investigator blinded to the treatment group evaluated the tissue sections from three different areas for each animal.

### 4.7. F11 DRG Cell Culture

The F11 neuronal cell line, an immortalized hybrid of embryonic rat DRG and mouse neuroblastoma cells, was used for investigating the role of hyperglycemia in the DRG neurons. F11 cells were grown in Neurobasal Medium (NBM) containing the following supplements: B-27, Glutamine, Albumex, and NGF-75. The cells were then divided into two groups: Normal Glucose (NG) and High Glucose (HG). The NG group served as our control and it was grown in NBM with supplements. The HG group was grown in NBM with supplements and an extra 25 mM glucose. NG and HG groups were both harvested after overnight exposure to normal or glucose media.

### 4.8. Statistical Analysis

A one-way analysis of variance (ANOVA, parametric) was used for the group comparisons and Bonferroni’s multiple comparison tests in post hoc analysis was followed. Furthermore, time dependent changes in outcomes were assessed while using repeated measures analysis of variance (rANOVA). All of the statistical analysis was accomplished using Systat 13. A *p* value of < 0.05 was counted as being significant. The results are demonstrated as mean ± SEM. Eight to ten animals per group were used for the animal behavior experiments, and they were repeated twice.

## 5. Conclusions

The results from this study suggest that the elevation of inflammatory mediators, including HMGB1 in DRG and spinal cord, are important in the development of painful neuropathy in Type 2 diabetes along with changes in histone acetylation. Our study further demonstrates that the increased levels of HMGB1, TLR4, and NLRP3 in DRG of the diabetic animals with PDN can be ameliorated by treatment with the HMGB1 inhibitor, GLC. This study also suggests that increased histone acetylation in the spinal cord and DRG neurons can be alleviated by HMGB1 inhibitor through the decreased expression of CXCR4, pJNK, and TRPC6 in the DRG and spinal cord respectively with a reduction in pain in diabetic animals. Hence, HMGB1 plays an important role in the painful neuropathy of Type 2 diabetic animals and it might exhibit a unique target for the treatment of this debilitating condition.

## Supplementary Materials

Supplementary Materials can be found at https://www.mdpi.com/1422-0067/21/3/881/s1.

## Abbreviations

DRG	dorsal root ganglia
HMGB1	high mobility group box 1
CXCR4	chemokine C-X-C motif receptor 4
RAGE	receptor for advanced glycation end products
TLR4	Toll-like receptor 4
NLRP3	Nod-like receptor protein 3
JNK	c-Jun N-terminal kinases
ZDF	Zucker diabetic fatty
GLC	Glycyrrhizin
H3K9ac	Histone 3 Lysine (K) 9 acetylation
