# Supplementary material for: High-Mobility Group Box 1 Protein Signaling in Painful Diabetic Neuropathy
Source: Int J Mol Sci. 2020 Jan 30;21(3):881. doi: 10.3390/ijms21030881 (PMC7036925; doi:10.3390/ijms21030881)
Supplement: Supplementary file 1 [file ijms-21-00881-s001.pdf]

Supplementary Figures

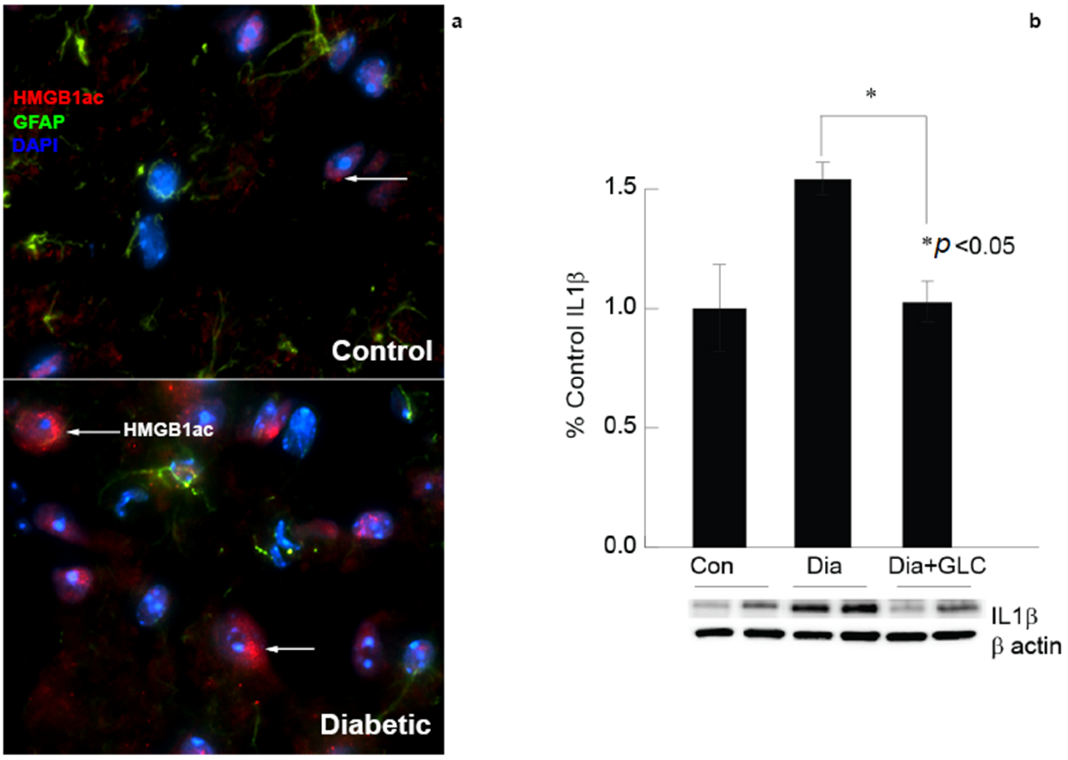

Figure S1. Increased HMGB1 acetylation and IL1β in the spinal cord dorsal horn of diabetic animals with painful neuropathy. (**a**) Ob/ob diabetic animals exhibited increased acetylation of HMGB1 in the neuronal and non-neuronal cells of spinal cord dorsal horn compared control group. (**b**) Increased IL1β expression in the spinal cord dorsal horn of ZDF Type 2 diabetic animals which was ameliorated by GLC treatment.

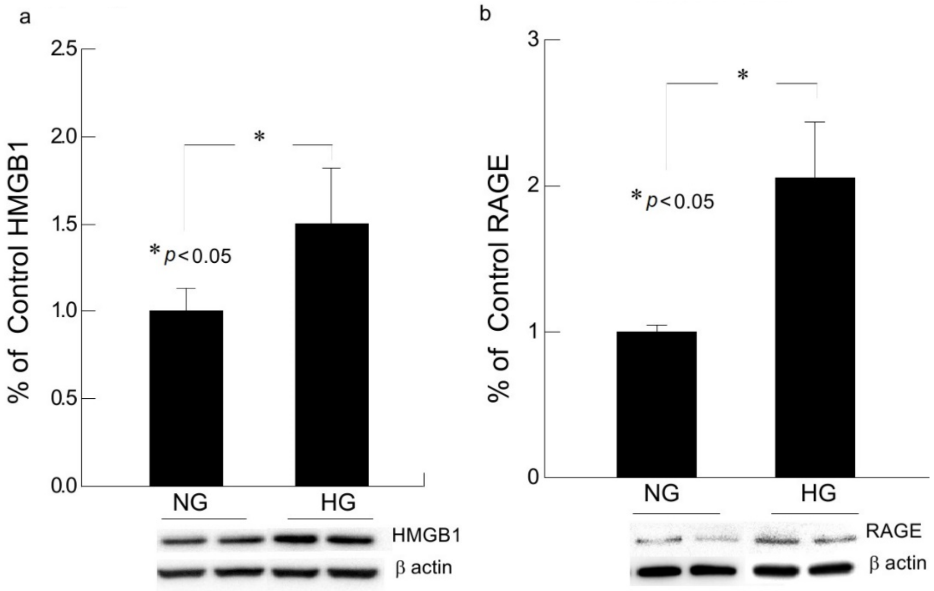

Figure S2. Increased expression of HMGB1 and RAGE in hyperglycemic DRG neuronal cell line. Overnight glucose exposure (25 mM) increased the expression of HMGB1and RAGE in F11 DRG cell line when compared with normoglycemic condition.

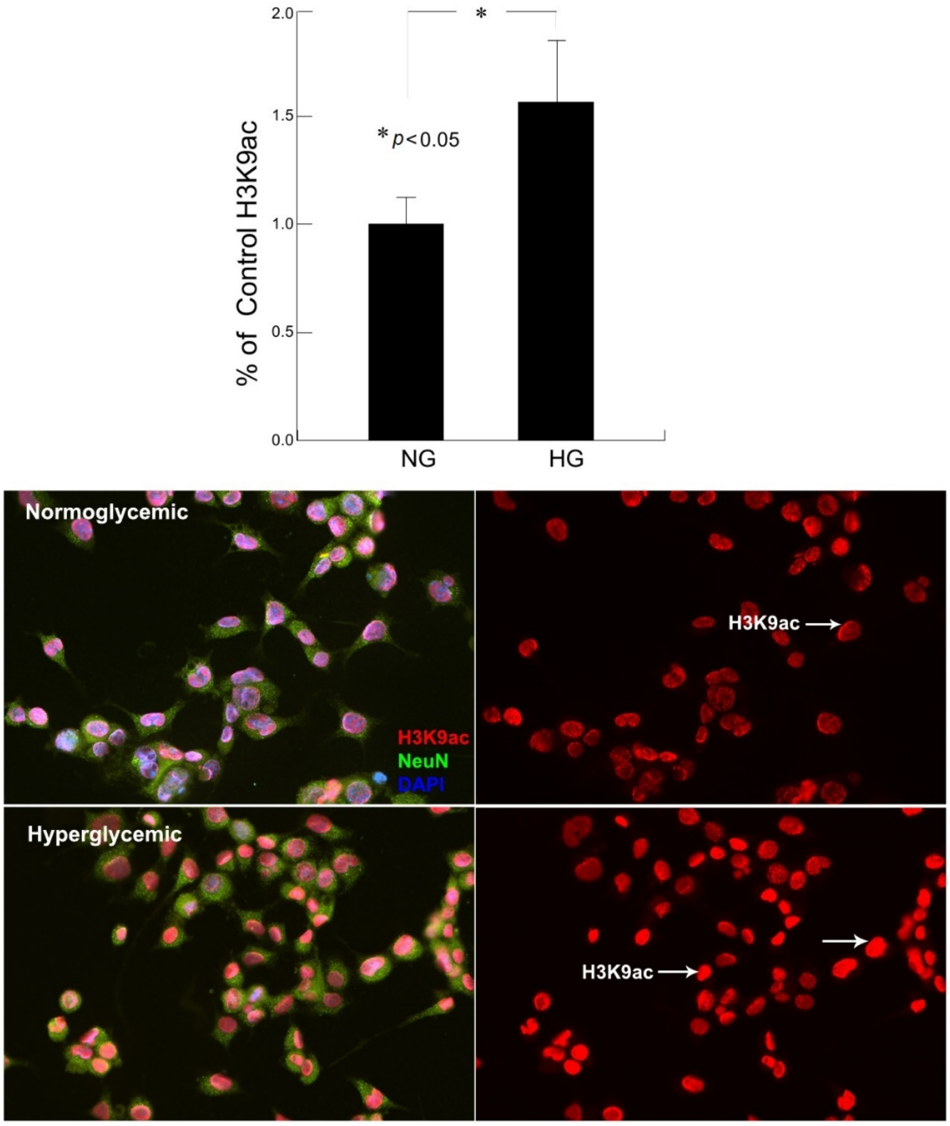

Figure S3. Enhanced H3K9 acetylation in hyperglycemic DRG neuronal cell line. Overnight exposure to 25mM glucose demonstrated increased H3K9 acetylation in hyperglycemic DRG neuronal cell line by immunocytochemical analysis.

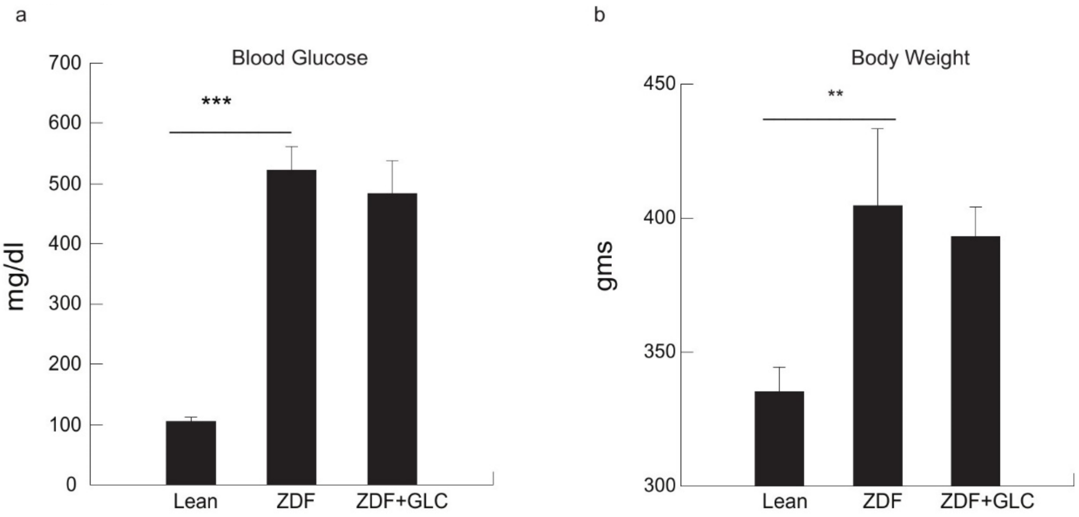

Figure S4. Weight and blood glucose levels in lean control and ZDF animals. ZDF diabetic animals gained weight significantly (** $p < 0.01$) 6 weeks after the onset of diabetes compared to lean control animals (**b**). Animals treated with GLC (ZDF+GLC) showed no change in blood glucose levels compared to diabetic only (ZDF) groups, whereas ZDF animals showed significant increase in blood glucose levels compared to the control animals (Lean; *** $p < 0.005$) as measured 6 weeks post-diabetes (**a**).
